# On the definition and identifiability of the alleged “hiatus” in global warming

**DOI:** 10.1038/srep16784

**Published:** 2015-11-24

**Authors:** Stephan Lewandowsky, James S. Risbey, Naomi Oreskes

**Affiliations:** 1 School of Experimental Psychology and Cabot Institute University of Bristol, 12A Priory Road, Bristol, BS8 1TU, United Kingdom; 2 School of Psychology, University of Western Australia, 35 Stirling Highway, Crawley, WA 6009 Australia; 3 CSIRO Oceans and Atmosphere, Box 1538, Hobart, Tasmania 7001 Australia; 4 Department of the History of Science, Science Center 371 Harvard University, 1 Oxford Street, Cambridge, Massachusetts 02138, USA.

## Abstract

Recent public debate and the scientific literature have frequently cited a “pause” or “hiatus” in global warming. Yet, multiple sources of evidence show that climate change continues unabated, raising questions about the status of the “hiatus”. To examine whether the notion of a “hiatus” is justified by the available data, we first document that there are multiple definitions of the “hiatus” in the literature, with its presumed onset spanning a decade. For each of these definitions we compare the associated temperature trend against trends of equivalent length in the entire record of modern global warming. The analysis shows that the “hiatus” trends are encompassed within the overall distribution of observed trends. We next assess the magnitude and significance of all possible trends up to 25 years duration looking backwards from each year over the past 30 years. At every year during the past 30 years, the immediately preceding warming trend was always significant when 17 years (or more) were included in the calculation, alleged “hiatus” periods notwithstanding. If current definitions of the “pause” used in the literature are applied to the historical record, then the climate system “paused” for more than 1/3 of the period during which temperatures rose 0.6 K.

“There was no such thing as the Scientific Revolution, and this is a book about it.”

—Steven Shapin, 1996, *The scientific revolution*. University of Chicago Press.

In the public sphere, the claim that global warming has “stopped” has long been a contrarian talking point^1^,^2^. After being confined to the media and internet blogs for some time, this contrarian framing eventually found entry into the scientific literature^3^,^4^, which is now replete with articles that address a presumed recent “pause” or “hiatus” in global warming^5^. The “hiatus” also featured as an accepted fact in the latest assessment report of the IPCC^6^. Despite its widespread acceptance in the scientific community, there are reasons to be skeptical of the existence of the “hiatus”^5^.

Recently, possible artifacts in the global surface temperature record have been noted which, when corrected, suggest that there is little evidence for a “hiatus” relative to the long-term trend used by the IPCC^7^. In addition, multiple other indicators such as ocean heat content point to continued warming^8^,^9^,^10^.

In this article, we show that even putting aside possible artifacts in the temperature record, there is no substantive evidence for a “pause” or “hiatus” in warming. We suggest that the use of those terms is therefore inaccurate. Because this conclusion appears to contradict the IPCC’s explicit endorsement of the “hiatus”, it is important to differentiate between the different ways in which the term “pause” or “hiatus” has been motivated and used in the recent climatological literature.

Research on the “hiatus” has been couched within at least 4 distinct research questions: (1) Is there a “pause” or “hiatus” in warming? (2) Has warming slowed significantly compared to the long-term trend? (3) Has warming lagged behind model-derived expectations? (4) What are the physical mechanisms responsible for the “hiatus”? Here, we are exclusively concerned with the first question: Is there, or has there recently been, a “pause” or “hiatus” in warming? We focus on this question because it is ineluctably tied to the contrarian claim that global warming has “stopped”, which has demonstrably affected the political and media landscape^3^ as well as, arguably, the scientific community^4^. The question whether there is a “pause” in global warming can be readily tested: Standard dictionary definitions of the words “pause” or “hiatus” imply that a process has been suspended or interrupted. It follows that for the notion of a “hiatus” in global warming to be scientifically well-founded, there must either be a demonstrable and statistically-relevant absence of any trend in global mean surface temperature (GMST) during the time period that is considered relevant or, minimally, the observed trend must differ in a statistically identifiable way from the historical record.

Our focus on the question whether there is a “hiatus” or “pause” implies that we do not address two related issues: First, we are not concerned with the differences, if any, between climate model projections and observed GMST trends. We have addressed the issue whether or not warming has lagged behind model-derived expectations elsewhere^11^, and this issue has no bearing on the existence of a “hiatus”. Second, we are not concerned with the underlying physical processes that may explain fluctuations, whether positive or negative, in GMST. This is again a different question, which is interesting in its own right but has no bearing on the existence of a “hiatus”.

We examine the status of the “hiatus” in three steps: First, we compile an inventory of operationalizations of the “hiatus” in the existing scientific literature and ask whether they converge on a consistent definition. Second, we ask whether the rate of temperature change during the “hiatus”, as it is operationalized in the literature, differs meaningfully from the set of rates for equivalent trend lengths observed during the era of modern climate change. This comparison is essential because *any* trend will exhibit periods of statistical insignificance when the sample size (i.e., number of years considered) is small: The existence of the presumed “hiatus” thus cannot be ascertained without a historical comparison to other comparable trend durations at earlier times during which warming was consensually thought to be present. Finally, for the same reason, we ask whether the duration of periods in which there is no significant warming has changed during the presumed “hiatus” relative to the rest of the modern period.

## Results

### There is no agreed “hiatus” period in the scientific literature

We catalogued a corpus of peer-reviewed articles published between 2009 and 2014 that specifically addressed the presumed “hiatus” in global warming. Table 1 shows that the term “hiatus” was used more than 550 times in this corpus, and the word “pause” in excess of 70 times.

**Figure 1:**
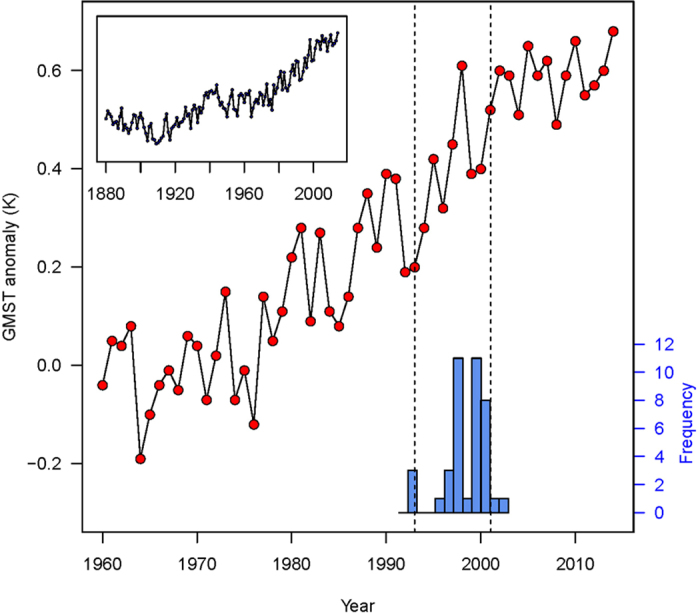
Global mean surface temperature (GMST) anomalies estimated by NASA’s Goddard Institute for Space Studies (GISS) data set (40 http://data.giss.nasa.gov/gistemp/, all analyses based on dataset downloaded on 17 January 2015). The histogram at the bottom represents the distribution of presumed start years for the presumed “hiatus” in the corpus of articles (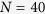; see Table 1) considered for this analysis. The vertical lines represent the 5th (1993) and 95th (2001) percentile, respectively, of presumed starting years for the “hiatus”. The small inset shows the overall historical temperature anomalies recorded since 1880.

Many articles assumed that the “hiatus” commenced around 1998, at which time temperature anomalies were considerably above the long-term trend. There is, however, considerable heterogeneity in published onset times, with the range spanning a decade (1993–2003). Similarly, there is considerable heterogeneity in the presumed duration of the “hiatus” across the same corpus of articles, with a range 10–20 (median 13 years, 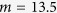, 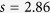). For each article, we took the duration to be the number of years since the assumed onset of the “hiatus” to the end of the period being analyzed. This constitutes a lower bound on the presumed duration of the “hiatus” as some authors may have presumed that the “hiatus” was ongoing at the time they published an article. Figure 1 shows the modern global temperature data together with a histogram of the distribution of presumed onset times of the “hiatus” derived from the corpus.

The heterogeneity in onset and duration raises the possibility that the use of the term “hiatus” departs from normal scientific practice, which strives to define phenomena on the basis of clear and generally accepted criteria. The heterogeneity may be explained by the supposition that authors defined the “hiatus” retrospectively, via an ad hoc analysis of the recent trend leading up to the time of writing, rather than on the basis of a priori criteria. This apparent lack of clear and a priori criteria must be of concern in the statistical environment in which the “hiatus” has unfolded, which is known to be sensitive to the particular choice of start and end points that define short-term trends and the comparison baseline^12^.

### The “hiatus” is an unexceptional fluctuation

**Figure 2:**
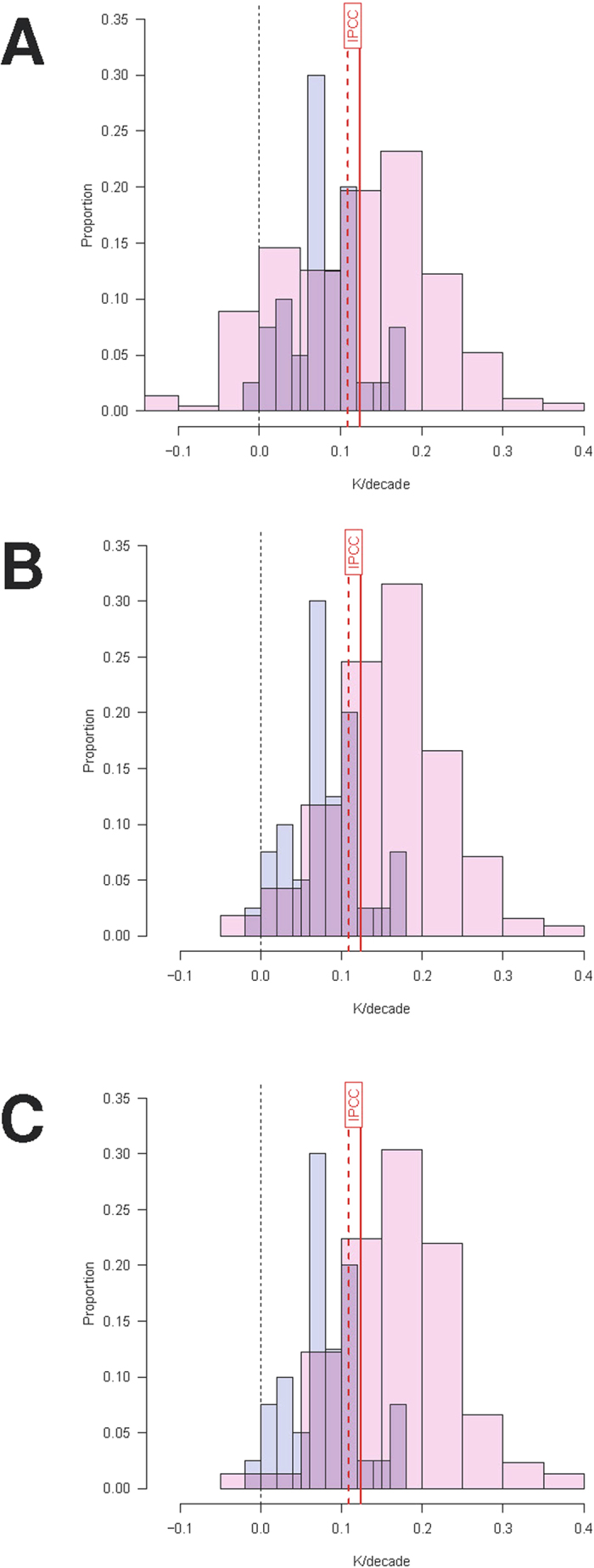
(**A**) distribution of observed decadal temperature trends (GISS) within the “hiatus” windows defined by the corpus of articles considered for this analysis (blue), compared to the distribution of all possible temperature trends from 1950 till 2012, the reference period used by the IPCC to establish the long-term warming trend (pink). (**B**) same distribution of temperature trends within the “hiatus” windows (blue) compared to the distribution of all possible temperature trends from 1964 till 2012 (pink). The year 1964 is the lower bound for the 95% confidence interval of a recent change-point analysis that sought to identify the onset of modern global warming. (**C**) same distribution of temperature trends within the “hiatus” windows (blue) compared to the distribution of all possible temperature trends from 1976 till 2012 (pink). The year 1976 is the upper bound for the 95% confidence interval of a recent change-point analysis that sought to identify the onset of modern global warming. In all panels, the distribution of all possible trends is obtained by computing all trends of a given duration from all possible years within the time period considered. The duration of trends is weighted by the propensity of presumed “hiatus” durations in the corpus. Thus, each 10-year trend is replicated 8 times (as 8 articles in the corpus presumed the “hiatus” to extend over 10 years), each 11-year trend 5 times, and so on. See Table 1 for details of the distribution of presumed “hiatus” durations in the corpus. The vertical red lines in each panel represents the long-term trend (1951–2012) that was used by the IPCC in their Fifth Assessment Report as a benchmark for comparison with the “hiatus.” The solid line is for the GISS dataset^40^ analyzed here, and the dashed line is the same long-term trend using the UK Met Office’s HadCRUT4 data set^39^ used by the IPCC.

If the definitions of the presumed hiatus are highly variable, with many different time periods proposed in the literature, how can we determine whether or not there is one? In order to answer this question, we compared the distribution of decadal warming trends during the “hiatus”—as defined by the articles in the corpus—against the distribution of all possible trends that have been observed during the period of modern global warming. The results are shown in Fig. 2, using three different onset dates for global warming.

The question of when, precisely, greenhouse-driven warming began to be observable against background natural variability is itself contested. An early review^13^ that examined the literature back to 1824 finds that scientific concern about global warming arose as early as 1938. Every decade since then has seen increased scientific attention and concern^13^, although no consensual onset date for global warming has been identified. Figure 2 therefore uses three different onset dates for the computation of all possible trends. Panel A uses the period 1951–2012, which was used by the IPCC in AR5 as the long term trend against which to define the “hiatus”^6^. Panel B uses 1964 as the onset of modern global warming, whereas Panel C uses 1976. Those two years are two standard deviations (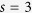) below and above, respectively, of the best estimate (1970) of the onset of modern global warming in the GISS data set reported in a recent change-point analysis^14^. Panels B and C therefore approximate the lower and upper bound, respectively, of the 95% confidence interval for the onset of modern global warming by the change-point measure. All panels include data through 2012 because many of the articles in the corpus were written when the latest available data were for 2012 (or even earlier). (See the Online Supplementary Material for an extension of our analysis to the entire instrumental record.)

To permit a commensurable comparison, in all panels the distribution of all possible trends has the same propensity of trend durations as the “hiatus” in the corpus. Thus, each possible 10-year trend is replicated 8 times (as 8 articles in the corpus presumed the “hiatus” to extend over 10 years), each 11-year trend 5 times, and so on as determined by the propensity of trend durations in the corpus. The distribution of trend durations is therefore identical between the two histograms in each panel.

Figure 2 demonstrates that, although the distribution of trends during the “hiatus” is shifted downward compared to the overall distribution of trends of the same durations, the “hiatus” distribution falls within the overall envelope of historically observed trends. For the IPCC base period (1951–2012; Panel A) there is little discernable difference between the two distributions. For the two years that bracket the most likely change-point onset of the modern warming period (Panels B and C), the “hiatus” distribution is more clearly offset towards the lower end but it is by no means unusual or extreme.

Moreover, for nearly 15% of imputed “hiatus” trends (5 out of 40 articles in the corpus), the warming exceeded the long-term trend used by the IPCC (1951–2012; vertical red lines in Fig. 2). Similarly, nearly 20% of operationalizations (7/40) referred to a period during which temperatures increased significantly (i.e., 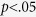 in OLS regression), which is not consistent with a “hiatus.”

The results in Fig. 2 show that all operationalizations of the “hiatus” in the literature are unexceptional in the context of equivalent-length trends in the record of modern global warming. At most, the operationalizations in the literature support the conclusion that the rates of warming over some recent intervals have been toward the lower end of the historically-observed surface temperature record. However, they do not support the conclusion that there is a “pause” or “hiatus” in the warming.

### The “hiatus” has always been there when sample size is small

We next analyzed the GMST data from all possible different vantage points (end years looking back in time) to examine whether a scientist in, say, 2014 or 2010 would have been justified in accepting the existence of a “hiatus” in warming relative to what would have been detectable at any other prior point in time.

**Figure 3:**
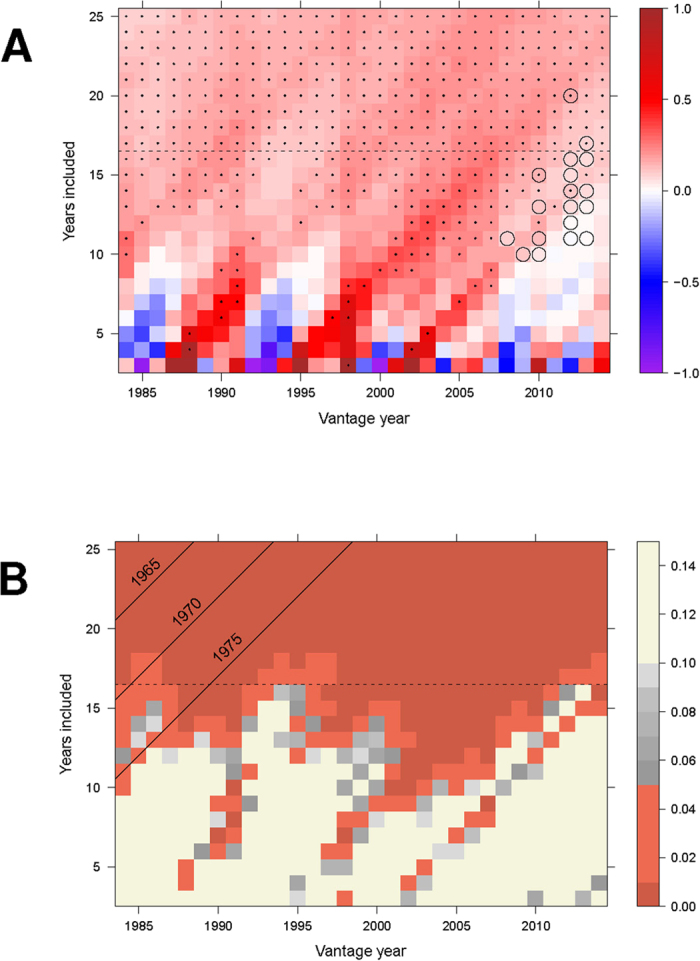
(**A**) Observed magnitude of temperature trends (GISS, K/decade) as a function of vantage year and the number of years included in the computation of the trend. Trends are capped at ±1 *K* for plotting. For each vantage year, trends are computed for all possible windows between 3 and 25 years duration, all of which end with the particular vantage year. The dots indicate which trends are significant (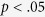) in an ordinary least squares analysis of annual means, and the horizontal dashed line indicates the number of years that must be included (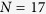) for the trend to be significant from all vantage points. The open circles identify combinations of onset and duration that have been used to identify the “hiatus” by articles in the corpus. Multiple articles may contribute to a given circle. The Online Supplementary Material shows that the basic conclusions are unaffected by consideration of autocorrelations, although an additional 2 years are required to reach significance for all vantage points across the entire 30-year period. (**B**) Level of statistical significance for trends (GISS, K/decade) as a function of vantage year and the number of years included in the computation of the trend. Trends that are clearly non-significant (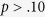) are shown in beige, those that approach significance (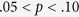) are shown in shades of gray, and significant trends (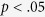) are shown in shades of terracotta. The diagonal lines identify calendar years that contribute to the analysis. Any observation in the grid that lies to the Southeast of a given line includes only observations from the stated year onward, and any observation to the Northwest also includes earlier years. The observation in the top-left corner is 1960 (i.e., looking backward 25 years from 1984).

Figure 3(A) shows the warming trends that were observable, given the available data at the time, for any vantage point between 1984 and 2014 (horizontal axis). For each vantage point, between 3 and 25 years were included in the trend calculation (vertical axis). The Online Supplementary Material extends this analysis to even longer time scales. Timescales of at least 17 years are known to be necessary for noise reduction and detection of a signal^15^.

Figure 3(A) shows that at every year (vantage point) during the past 30 years, the immediately preceding warming trend was always significant when 17 years (or more) were included in the calculation (dots denote 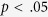). Figure 3(B) presents the same data using a ternary classification of *p*-values for the linear trend into non-informative (beige), partially informative but not conventionally significant (gray), and significant (terracotta). This panel also includes three diagonal lines that identify the earliest calendar year included in the analysis. Thus, any observation to the Southeast of the line labeled “1975” only includes observations later than that, and so on for the other two lines. The observations to the Northwest of “1965” go back to 1960 (top-left corner; looking back 25 years from 1984 inclusive).

The large beige area in Panel B highlights the well-known fact that when sample size is small, statistical power is often insufficient to differentiate signal from noise. Conversely, the large terracotta area highlights the fact that when power is sufficient, the warming signal has been detectable at any point during the last 30 years, irrespective of vantage point. When one extends the period looking backwards in time, the warming trend is always significant, and the most recent vantage point(s) do not differ systematically from earlier vantage points. It follows that the data do not permit identification of a “pause” or “hiatus” during the last 10–20 years. Significantly, this conclusion is unaffected by the choice of year taken to represent the onset of modern warming (i.e., areas to the Southeast of all 3 diagonal lines in Figure 3(B) permit the same conclusion). The conclusion is also unaffected by the choice of the year during which the “pause” was examined (i.e., the vantage point).

Conversely, if one uses shorter time periods of analysis, one can find many “pauses.” Using the operationalizations found in the corpus (mean duration 13.5 years), and a null hypothesis of no warming, we find that the climate “paused” strikingly often during the last 30 years. During that period, the 14-year trend escaped significance 10 times and the 13-year trend 13 times, suggesting that a “pause” occupied between 30% and 43% of a time period during which the climate warmed 0.6 K overall (Fig. 1). If the duration of the defined “hiatus” drops to below 12 years—which applies to 13 out of 40 articles (i.e., 32.5%) in the corpus—then almost everything is a “hiatus”, as signified by the preponderance of beige for trends of this duration in Fig. 3(B). Anyone making a “hiatus” claim of this duration will almost always find one, not because something new and different is happening, but because of the fundamental fact that small sample sizes provide insufficient statistical power for the detection of trends. Thus, a third of the articles in the corpus either presumed that the climate has nearly always “paused” during the last 30 years (rendering the term meaningless), or they inconsistently highlighted only one of many events that would qualify with their definition.

These results have been replicated using a variety of additional methods that incorporate autocorrelations in the time series (see the Online Supplementary Material). The results are not sensitive to the trend detection methods employed, and they are also not sensitive to the choice of GMST data set (see the Online Supplementary Material).

We conclude that the evidence does not support the notion of a “pause” or “hiatus” as an identifiable phenomenon that is implied by standard dictionary definitions and common understandings of these terms.

## Discussion

We recognize that our claim that there is no “hiatus” will be controversial, particularly in light of the widespread embrace of the “hiatus” in public and scientific discourse. Therefore, it is important to clarify what we are *not* claiming. First, and perhaps most important, we do not argue against the merit of research on decadal-scale variation in the climate. On the contrary, the numerous articles on the “hiatus” have contributed to our understanding of what drives decadal fluctuations in climate, including for example its seasonal aspects^16^. Notably, none of the articles in our corpus indicate that they expect the “hiatus” to continue indefinitely, implying that they do not support some public interpretations that recent fluctuations in the GMST undermine the scientific basis for understanding anthropogenic climate change^17^.

Second, our exclusive focus on GMST relative to the null hypothesis of no trend was mandated by our goal to examine the notion of a “pause” or “hiatus” with respect to the observations alone. It does not follow that global trends constitute the only—or even preferable—level of analysis for the climate system.

Third, we do not explicitly address the question whether warming has slowed significantly during the presumed “hiatus” period, although we have suggested elsewhere that it has not^5^. In confirmation, a recent change-point analysis of GMST has shown that there is no statistically-identifiable change in warming trend after the 1970s^14^.

Fourth, our analysis does not speak to the apparent or presumed discrepancy between model projections and GMST trends. Research on this question has identified several effects and variables that can reconcile apparent differences between modeled and observed temperatures during the recent fluctuation, such as model-versus-observed differences in the phasing of internal variability^11^,^18^,^19^, systematic errors in some of the external forcings used in CMIP5 simulations^20^,^21^, and incomplete coverage and quality of observations^7^.

Finally, our demonstration that the “hiatus” is statistically indistinguishable from previous fluctuations has no bearing on the question of the physical causes of fluctuations in surface temperature trends. Such fluctuations can be due to internal variability alone^12^,^22^,^23^, or they may involve variations in external forcings on the climate system such as solar cycles or volcanic eruptions, or both^24^,^25^,^26^. We have no commitment to a particular causal model of those fluctuations.

## Conclusions

We have shown that there is a wide range of different operationalizations of the “hiatus” in the literature. For none of these operationalizations is the rate of temperature change meaningfully different from the set of rates of equivalent trend lengths over the modern period. That is, the “hiatus”, however defined, is not unusual or unprecedented^27^. Further, the duration of periods over which trends must be extended to generate significant warming trends has not changed noticeably in the “hiatus” periods relative to the rest of the modern warming period. We conclude that there is no “hiatus”, and neither has the climate system “paused.”

Our conclusion raises at least two questions. First, why has so much research been directed at the “hiatus” when it does not exist? We have addressed the likely reasons for this in detail elsewhere^4^. The notion of a “pause” or “hiatus” demonstrably originated outside the scientific community^3^, and it likely found entry into the scientific discourse because of the constant challenge by contrarian voices that are known to affect scientific communication and conduct^4^,^28^,^29^.

The second question pertains to the broader implications of this apparent discord between data and the discussion in the literature. We suggest that discussing climate change using the terms “pause” or “hiatus” creates notable hazards for the scientific community.

Adoption of the terms “hiatus” or “pause” is not inconsequential because the way in which environmental issues are linguistically and semantically framed contributes crucially to public (mis-)understanding^30^. Scientists may argue that when they use the terms “pause” or “hiatus” they know—and their colleagues understand—that they do not mean to imply that global warming has stopped. Indeed, the use of scare quotes in some articles (Table 1) is clearly intended to imply this. The problem, however, is that these terms have vernacular meanings, and when scientists use a term from the public vernacular to describe a feature of science, confusion results when the vernacular term is not an appropriate description of that feature. This misunderstanding may be particularly acute in this instance because the terms “pause” and “hiatus” originated as contrarian talking points^3^,^4^. Hence, we argue that scientists should use the term that most appropriately describes what they are studying. In the present case, that implies the use of “fluctuation”, not “hiatus,” because when scientists use the term “hiatus”, this sends a confusing and potentially misleading message to the public. Scientists might tacitly understand that global warming continues notwithstanding the alleged “hiatus”, or they may intend the “pause” to refer to differences between observed temperatures and expectations from theory or models, but the public is not privy to that tacit understanding.

Moreover, acceptance and use of scientific propositions carries ethical implications and responsibility^31^,^32^. Some philosophers argue that holding a belief without sufficient “warrant”—i.e., without support by strong evidence—engenders a moral hazard^33^. An important element of this argument is that any belief, no matter how innocuous or inconsequential, creates the enabling conditions for similar and related beliefs. Any belief or opinion thus contributes to shaping an epistemological landscape, which in turn implies a responsibility—or when the belief is unwarranted, a moral hazard—for “downstream” intellectual consequences. Specifically, if unwarranted acceptance of a “hiatus” in global warming contributed to the delay of political action to mitigate climate change, with potentially adverse consequences on innocent parties, then the scientific status of the “hiatus” could become a matter not just of science and philosophy, but also ethics and even law. Lest one consider such a potential hazard remote, the legal aftermath of the earthquake in L’Aquila, Italy, which embroiled scientists in charges of manslaughter for their alleged failure to warn the community^34^,^35^, vividly illustrates the legal and moral hazards that are incurred when the public is not adequately informed of the full envelope of identifiable risks arising from scientific findings. In this context, it is notable that in a blind expert test, the notion that global warming has “stopped” was found to be misleading in light of the data^5^.

Those hazards can be largely avoided in this case by clear communication, which includes (although to be sure is not limited to) avoiding the unsubstantiated use of “pause” or “hiatus” when referring to fluctuations of GMST about the longer-term warming rate.

## Methods

### Corpus of articles

Table 1 summarizes the corpus of 44 articles that explicitly addressed the “hiatus”, either by seeking an explanation or by reconciling it with model output. Only articles addressing global (as opposed to regional) temperatures were included. Articles were sourced by the authors with the help of a number of other researchers and climate experts who are conversant with the current literature.

For each article, the table records the number of times that keywords such as “slowing”, “pause”, or “hiatus” occurred in the text. Occurrences in the reference section, in running heads, or in metadata were not counted. All forms of the stem were accepted; e.g., “slow”, “slowed”, “slowing”, and so on. Note that Crowley *et al.*^36^ used another term, namely “plateau”, 13 times. In addition, the word “stop” appeared 4 times in two articles^37^,^38^. Wherever a number is put into quotation marks (e.g., “1”) this refers to the number of times the term was put into “scare quotes,” implying that the term was not necessarily accepted by the author. When scare quotes were used together with unquoted occurrences, those other occurrences are provided after the “+” symbol.

Where applicable, the table also presents a quotation (usually from the abstract or first paragraph) that was judged to be indicative of the “framing” of the article. Citations or acronyms (or clauses not relevant to the meaning) in the quotation are omitted and replaced by…. When the quotation is absent for an article, a clear identification of framing was not possible. The *Focus* column indicates whether the “hiatus” was discussed primarily with respect to the observations (O) or with respect to the match between models and observations (M), or both (OM). The *Data* column indicates which data set was used by the study, where H = HadCRUT4^39^; G = GISS^40^; N = NCDC^41^; CW = Cowtan & Way^42^; C3 and C5 refer to CMIP3^43^ and CMIP5^44^ model ensembles, respectively; and “o” refers to other data sets.

The table also records the presumed onset of the “hiatus” as stipulated in each article (column labeled *From*) and the end of the “hiatus” (*To*). Concerning onset, articles sometimes use fuzzy terminology such as “first decade of 21st century” (interpreted to mean 2000–2009) or “2000s” (also taken to mean 2000–2009), or they contain several explicit and mutually incompatible onset times (in which case the first or more explicit one was taken as the article’s declaration of onset). Similarly, the presumed end of the “hiatus” sometimes remained unclear as it was often (but not always) the “present” or time of writing of the article. It was not always possible to unambiguously identify the last observation in the data set. Because of those potential ambiguities, a second independent reader who was blind to the purpose of the study audited, and confirmed, the values derived by the first author. Unambiguous identification of onset and duration proved impossible for 4 articles, and the main analyses are therefore based on 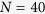. The corpus reported in Table 1 does not claim to be exhaustive; note, however, that the inclusion of further articles cannot reduce the range of onset times—it could only extend it.

The *Trend* column indicates if the trend in the observations (NASA’s GISS data set;^40^) was significant for the time period specified (* denotes 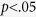) and whether it exceeded the IPCC’s long-term reference trend (1951–2012), denoted by >I. Entries in this column that are labeled NA are not included in the quantitative analysis because computation of the trend was prevented by ambiguity in the operationalization of the “hiatus.”

The table omits articles that did not address global mean surface temperature (GMST) but exclusively focused on other indicators such as ocean heat content or temperature^9^,^45^,^46^; sea level rise^47^; or wind^48^.

## Additional Information

**How to cite this article**: Lewandowsky, S. *et al.* On the definition and identifiability of the alleged “hiatus” in global warming. *Sci. Rep.* **5**, 16784; doi: 10.1038/srep16784 (2015).

## Supplementary Material

Supplementary Information

## Figures and Tables

**Table 1:** Summary of literature on the “hiatus”.

Article	From	To	Trend	“Slow”	“Pause”	“Hiatus”	Focus	Data
Allan^49^	2000	2012		4	—	—	OM	H, C5, o
	“…energy is continuing to accumulate in the oceans, despite the apparent recent slower rates of global surface warming compared with the late twentieth century and with climate model simulations.”							
Brown^50^	≃2001	2012		3	—	“1”	M	C5
	“A slowdown in the rate of warming in the early 21st century has increased interest in unforced decadal variability within the scientific community.”							
Chen^51^	≃2001	≃2010		17	—	15	O	o
	“The latter part of the 20th century saw rapid global warming as more heat stayed near the surface. In the 21st century, surface warming slowed as more heat moved into deeper oceans.”							
Clement^52^	2000	2013		1	5	“2”+8	O	o
	“A pause in global warming since 2000—a global warming ‘hiatus’—has opened up new questions about natural and human activity-driven (anthropogenic) effects on global mean trends in surface temperature.”							
Crowley^36^	1997 (2002)	2013	*	—	—	—	O	H, o
	“Stable global temperatures of the last 10–15 years have been a topic of considerable discussion.”							
Drijfhout^53^	≃2001	≃2010		2	—	11	O	G, H, o
	“…a slowing of the warming in the 2000s, even though atmospheric greenhouse gas concentrations continued to increase. This hiatus in warming may have been exaggerated by sampling errors [Cowtan and Way, 2014], but a significant slowdown is evident.”							
Easterling^54^	1998	2008		—	—	—	O	N, C3
	“Numerous websites, blogs and articles in the media have claimed that the climate is no longer warming, and is now cooling. Here we show that periods of no trend or even cooling of the globally averaged surface air temperature are found in the last 34 years of the observed record, and in climate model simulations of the 20th and 21st century forced with increasing greenhouse gases.”							
England^18^	2001	2013		2	—	27	O	G, C5
	“Despite ongoing increases in atmospheric greenhouse gases, the Earth’s global average surface air temperature has remained more or less steady since 2001.”							
Estrada^55^	late 1990s	2012		15	1	—	O	G, H, o
	“The warming of the climate system is unequivocal as evidenced by an increase in global temperatures by 0.8C over the past century. However, the attribution of the observed warming to human activities remains less clear, particularly because of the apparent slow-down in warming since the late 1990s.”							
Fyfe^56^	1993 (1998)	2012	* >I	2	—	“1”	M	H, C5
	“Recent observed global warming is significantly less than that simulated by climate models.”							
Fyfe^57^	1993	2012	* >I	—	—	1	M	H, C5
Goddard^58^	≃2003	≃2013		2	—	“1” + 7	O	o
	“The ‘global warming hiatus’—the fact that globally averaged air temperatures have not increased as quickly in the past decade as they have in previous decades—is a hot topic, so to speak.”							
Guemas^59^	2000	2010		26	7	3	O	o
	“Despite a sustained production of anthropogenic greenhouse gases, the Earth’s mean near-surface temperature paused its rise during the 2000–2010 period.”							
Haywood^60^	2002	2012		4	—	4	M	H, o
	“The slow-down in global warming over the last decade has lead to significant debate about whether the causes are of natural or anthropogenic origin.”							
Hawkins^37^	1998	2012	* >I	12	“2” + 12	—	O	H, o
	“The recent slowdown (or ‘pause’) in global surface temperature rise is a hot topic for climate scientists and the wider public.”							
Held^61^	1993	2012	* >I	—	1	10	M	H, o
	“A global climate model that factors in the observed temperature of the surface ocean in the eastern equatorial Pacific offers an explanation for the recent hiatus in global warming.”							
Huber^62^	≃1998	2012		3	—	“2” + 2	OM	H, CW, C, o
	“Global mean surface warming over the past 15 years or so has been less than in earlier decades and than simulated by most climate models.”							
Hunt^63^	1998	2010		—	—	22	O	H, o
	“Controversy continues to prevail concerning the reality of anthropogenically-induced climatic warming. One of the principal issues is the cause of the hiatus in the current global warming trend.”							
Kamae^64^	≃1998	2012		5	2	9	O	G, C5
	“This global-warming hiatus is a period characterized by a pause in global SAT increase, despite a continued increase in radiative forcing….”							
Kaufmann^65^	1998	2008		7	—	8	O	H, G, o
	“Given the widely noted increase in the warming effects of rising greenhouse gas concentrations, it has been unclear why global surface temperatures did not rise between 1998 and 2008.”							
Kosaka^66^	2001	2012		2	—	28	M	H, o
	“Despite the continued increase in atmospheric greenhouse gas concentrations, the annual-mean global temperature has not risen in the twenty-first century, challenging the prevailing view that anthropogenic forcing causes climate warming.”							
Lin^67^	“>decade”		NA	—	1	8	O	N, o
	“The recent global-warming hiatus is attributed to a La Niña-like decadal cooling phenomenon over the eastern tropical Pacific Ocean.”							
Lovejoy^68^	1998	2013		“1” + 1	“10” + 21	“1”	M	G, o
	“More troubling, the models over-estimated the post-1998 El Ni 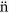 o global temperatures: they did not anticipate the ‘global slow-down’…, ‘hiatus’…, or ‘pause’....”							
Lu^69^	unspecified			2	—	“1” + 15	M	o
	“The global warming hiatus does not necessarily mean a hiatus in anthropogenic greenhouse gas forcing and forced climate change….”							
Macias^70^	2001	2013		1	—	“1” + 13	O	H
	“Global surface temperature has been increasing since the beginning of the 20th century but with a highly variable warming rate, and the alternation of rapid warming periods with ‘hiatus’ decades is a constant throughout the series.”							
Maher^71^	2001	2013		1	—	“1” + 104	M	G, C
	“The latest generation of climate model simulations are used to investigate the occurrence of hiatus periods in global surface air temperature in the past and under two future warming scenarios.”							
McGregor^72^	unspecified		NA	1	2	3	OM	G, o
Meehl^73^	2000	2009		—	—	34	M	o
	“There have been decades, such as 2000–2009, when the observed globally averaged surface-temperature time series shows little increase or even a slightly negative trend (a hiatus period).”							
Meehl^74^	2000	2009		1	—	79	OM	o
	“Globally averaged surface air temperatures in some decades show rapid increases (accelerated warming decades), and in other decades there is no warming trend (hiatus decades).”							
Meehl^19^	2000	2009		—	—	13	M	o
Meehl^75^	2000	2013		2	—	32	M	H, C5
	“The slowdown in the rate of global warming in the early 2000s is not evident in the multi-model ensemble average of traditional climate change projection simulations.”							
Palmer^76^	unspecified		NA	—	9	—	M	C5
Ridley^77^	“post 2000”			2	“1”	“1” + 2	O	o
	“Understanding the cooling effect of recent volcanoes is of particular interest in the context of the post-2000 slowing of the rate of global warming.”							
Risbey^11^	1998	2012		—	—	“1”	M	H, G, C5, CW
	“The differences between climate model forecasts and projections have come to prominence over interpretation of model simulations of recent temperature trends.”							
Santer^25^	1998	2012		4	—	“3”	O	C5, o
	“Despite continued growth in atmospheric levels of greenhouse gases, global mean surface and tropospheric temperatures have shown slower warming since 1998 than previously.”							
Schmidt^21^	1997	2013	*	2	—	—	M	G, CW, C5
	“Climate models projected stronger warming over the past 15 years than has been seen in observations.”							
Seneviratne^78^	1997	2012		4	“1” + 5	5	O	H, o
	“Observational data show a continued increase of hot extremes over land during the so-called global warming hiatus. This tendency is greater for the most extreme events and thus more relevant for impacts than changes in global mean temperature.”							
Sillmann^79^	1996	2010	* >I	1	—	“1” + 10	M	C5, o
	“The discrepancy between recent observed and simulated trends in global mean surface temperature has provoked a debate about possible causes and implications for future climate change projections.”							
Smith^80^	≃1998	2012		11	—	—	OM	H, G, N, C5
	“…it is now clear that the rate of warming has slowed substantially over the past 15 years or so and the observations are very much at the lower end of model simulations.”							
Solomon^81^	2000	2009		1	—	—	OM	H, G, N, o
Trenberth^38^	2000	2012		4	1	13	O	H, G, N, o
	“Global warming first became evident beyond the bounds of natural variability in the 1970s, but increases in global mean surface temperatures have stalled in the 2000s.”							
Trenberth^16^	1999 (2000)	2012	*	2	6	11	O	G, N, o
	“Although the 2000s are by far the warmest decade on record, the rate of increase of global mean temperature since 2000 has slowed....”							
Watanabe^82^	2000	2009		4	—	27	OM	H, G, C3, C5
	“The rate of increase of global-mean surface air temperature… has apparently slowed during the last decade.”							
Watanabe^83^	2000	2009		3	2	14	M	H, C3, C5
	“Reasons for the apparent pause in the rise of global-mean surface air temperature… after the turn of the century has been a mystery, undermining confidence in climate projections.”							
