# Supplementary material for: On the definition and identifiability of the alleged “hiatus” in global warming
Source: Sci Rep. 2015 Nov 24;5:16784. doi: 10.1038/srep16784 (PMC4657026; doi:10.1038/srep16784)
Supplement: Supplementary Information [file srep16784-s1.pdf]

Online Supplement for: On the operationalization and identifiability of the alleged "hiatus" in global warming

Stephan Lewandowsky
University of Bristol and University of Western Australia

James S. Risbey
CSIRO Oceans and Atmosphere, Hobart, Tasmania

Naomi Oreskes
Harvard University

Long-term trend

Figure S1 shows the effects of extending the timescales considered in the vantage-point analysis to include up to 60 years, and the entire instrumental record dating back to 1880. With the perspective provided by adding more vantage years, we see that short term trends (15 years or less) have always attained large magnitudes (of both signs). The figure shows that some of the medium term trends (30 years) can also include cooling in the past, but even those uniformly revert to warming trends when longer duration periods (greater than 50 years) are considered.

Autocorrelations

Climatological time series frequently exhibit autocorrelations, which render the significance levels of ordinary least squares regression (OLS) too liberal, thereby increasing the chances of a Type I error (i.e., detecting a trend when none is present). The IPCC recommends several possible solutions to the problem (Hartmann et al., 2013), two of which were explored here.

Generalized least squares

We replaced OLS with a generalized least squares (GLS) analysis in which the correlation among residuals was modeled using an AR1 process. Because estimation of autocorrelations is impossible or fraught with difficulty for short time series, we repeated the vantage-point analysis reported in Figure 3 in the main article with a minimum of 5 years included in the trend analysis. The results are shown in Figure S2. The pattern replicates the analysis in Figure 3 in the main article except that owing to the modeling of autocorrelations, now 19 (rather than 17) years are required to ensure significance at all vantage points.

Prewhitening of time series

An alternative means of dealing with autocorrelations is to apply OLS to the entire time series (in this case the period 1960–2014) and to adjust the raw time series using the overall time-lagged (i.e., AR1) correlation (Hartmann et al., 2013). Subsequent trend analysis on the adjusted time series is then done via OLS. This approach has the advantage that the autocorrelation is estimated once, using all available data, before trends of varying durations are computed by OLS, as in our main analysis underlying Figure 3 in the article.

The results of prewhitening are shown in Figure S3. The pattern replicates the analysis in Figure 3 in the main article except that owing to the prewhitening, now 19 (rather than 17) years are required to ensure significance at all vantage points.

Generalizing to other GMST data sets

We repeated the main landscaping analysis (Figure 3 in the article and Figures S2 and S3) using an alternative data set created by Cowtan and Way (2014). This data set is based on the U.K. Met Office's HadCRUT4 data set (Morice, Kennedy, Rayner, & Jones, 2012), but corrects for known coverage gaps in HadCRUT4, especially in the Arctic where warming is known to be most rapid. The data set by Cowtan and Way (2014) fills those gaps by mathematical interpolation (e.g., kriging).

The data of Cowtan and Way (2014) exhibit a slightly delayed onset of the upswing of temperature characteristic of the era of modern global warming compared to GISS. Accordingly, for this analysis we computed all possible trends from 1965 onward (as opposed to 1960 for GISS), to avoid "edge effects" for earlier vantage years.

Figure S4 shows the landscape of significance values for the data of Cowtan and Way (2014). The three panels in the figure report significance for ordinary least squares, generalized least squares, and prewhiting, respectively. The data support the same conclusions that we drew from analysis of GISS.

References

Cowtan, K., & Way, R. G. (2014). Coverage bias in the HadCRUT4 temperature series and its impact on recent temperature trends. *Quarterly Journal of the Royal Meteorological Society*, *140*, 1935-1944. doi: 10.1002/qj.2297

Hansen, J., Ruedy, R., Sato, M., & Lo, K. (2010). Global surface temperature change. *Reviews of Geophysics*, *48*, RG4004. doi: 10.1029/2010RG000345

Hartmann, D., KleinTank, A., Rusticucci, M., Alexander, L., Bronnimann, S., Charabi, Y., ... Zhai, P. (2013). Observations: Atmosphere and surface. In T. Stocker et al. (Eds.), *Climate change 2013: The physical science basis. contribution of Working Group I to the Fifth Assessment Report of the Intergovernmental Panel on Climate Change* (pp. 159–254). Cambridge, United Kingdom and New York, NY, USA: Cambridge University Press. doi: 10.1017/CBO9781107415324.008

Morice, C. P., Kennedy, J. J., Rayner, N. A., & Jones, P. D. (2012). Quantifying uncertainties in global and regional temperature change using an ensemble of observational estimates: The HadCRUT4 data set. *Journal of Geophysical Research: Atmospheres*, *117*, D08101. doi: 10.1029/2011JD017187

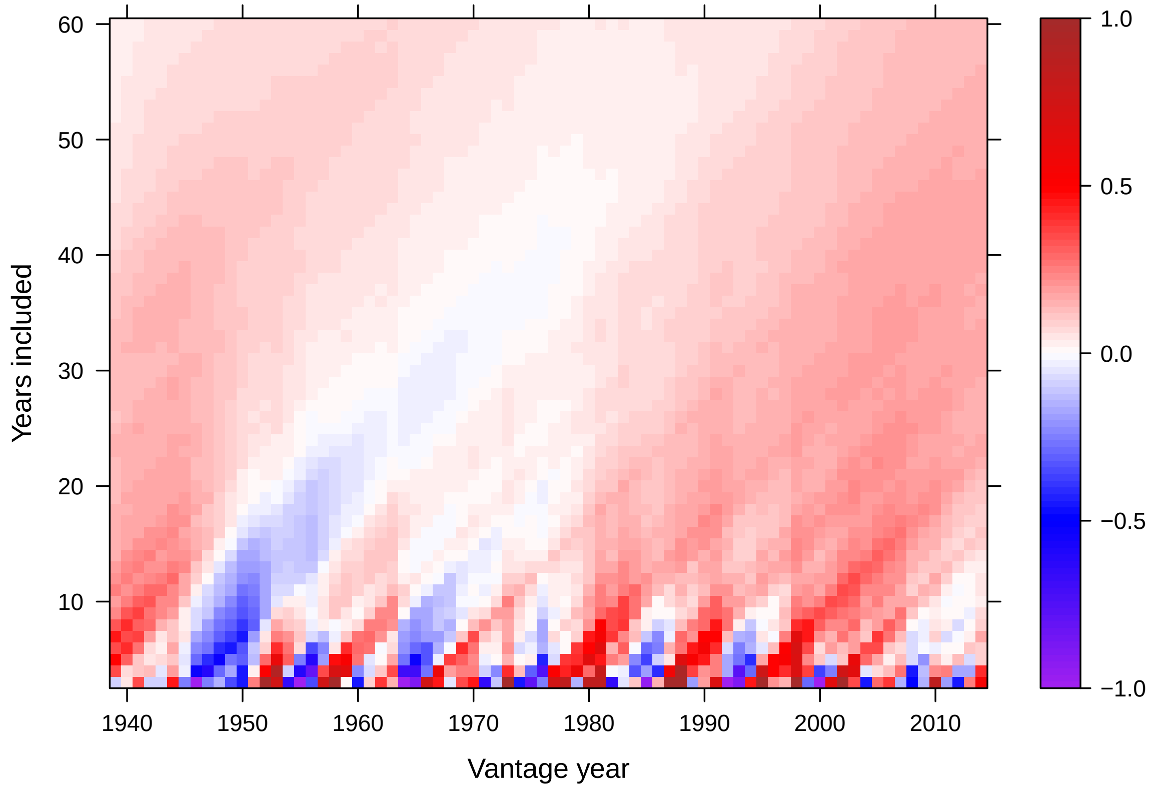

Figure S1. : Observed magnitude of global mean surface temperature (GMST) anomalies estimated by NASA's Goddard Institute for Space Studies (GISS) data set (Hansen et al., 2010; `http://data.giss.nasa.gov/gistemp/`, all analyses based on dataset downloaded on 17 January 2015) as a function of vantage year (1939–2014) and the number of years included in the computation of the trend by ordinary least squares analysis. Trends are expressed in K/decade and are capped at $\pm 1K$ for plotting. For each vantage year, trends are computed for all possible windows between 3 and 60 years duration, all of which end with the particular vantage year. The entire instrumental record (1880–2014) contributes to this analysis.

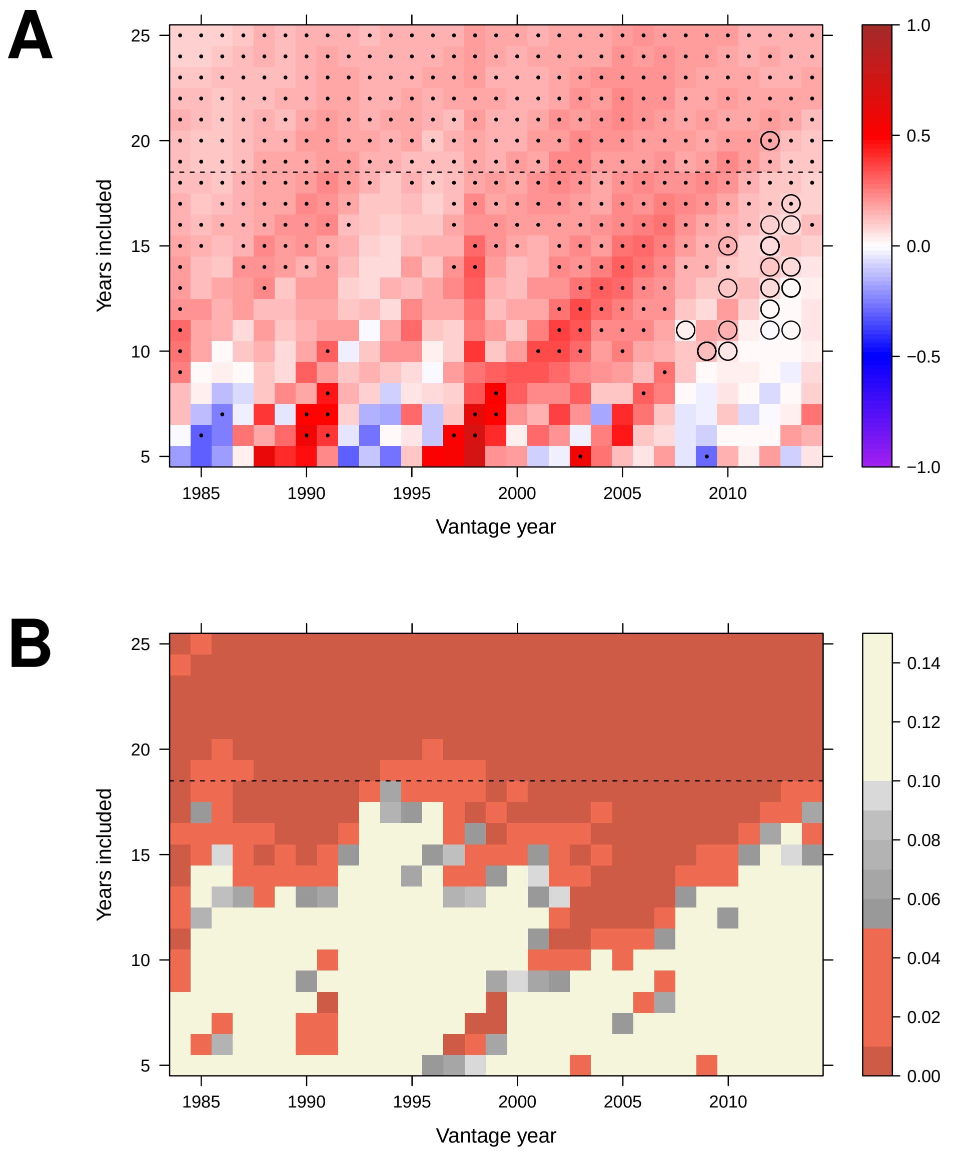

Figure S2. : **A**: Observed magnitude of temperature trends (GISS, K/decade) as a function of vantage year and the number of years included in the computation of the trend by generalized least squares analysis with autocorrelation among residuals modeled by an AR1 process as recommended by the IPCC (Hartmann et al., 2013). Trends are capped at $\pm 1K$ for plotting. For each vantage year, trends are computed for all possible windows between 5 and 25 years duration, all of which end with the particular vantage year. The dots indicate which trends are significant ($p < .05$), and the horizontal dashed line indicates the number of years that must be included ($N = 19$) for the trend to be significant from all vantage points. The open circles identify combinations of onset and duration that have been used to identify the "hiatus" by articles in the corpus. Multiple articles may contribute to a given circle. **B**: Level of statistical significance for trends (GISS, K/decade) as a function of vantage year and the number of years included in the computation of the trend as in panel A. Trends that are clearly non-significant ($p > .10$) are shown in beige, those that approach significance ($.05 < p < .10$) are shown in shades of gray, and significant trends ($p < .05$) are shown in shades of terracotta.

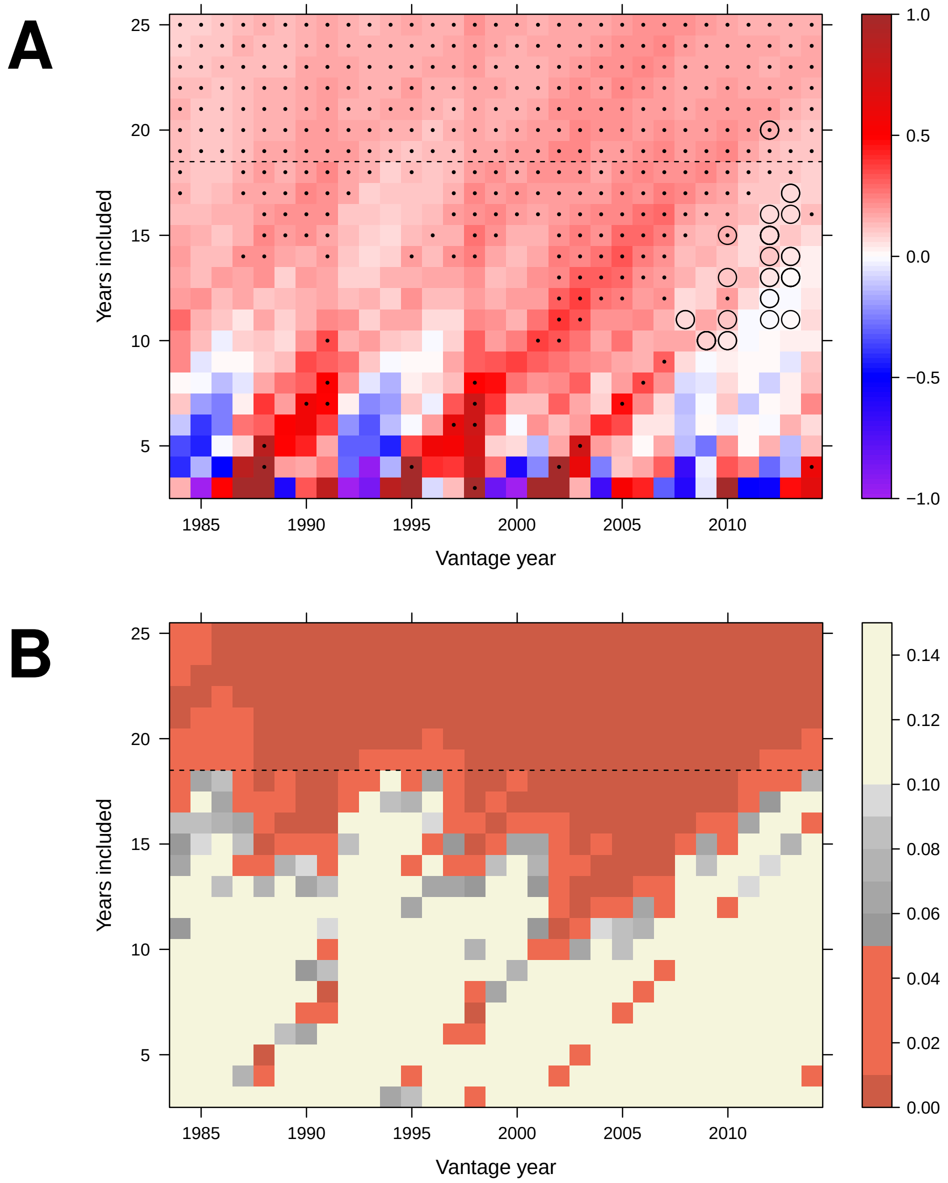

Figure S3. : **A**: Observed magnitude of temperature trends (GISS, K/decade) as a function of vantage year and the number of years included in the computation of the trend by ordinary least squares analysis on a prewhitened time series. See text for details on prewhitening. Trends are capped at $\pm 1K$ for plotting. For each vantage year, trends are computed for all possible windows between 3 and 25 years duration, all of which end with the particular vantage year. The dots indicate which trends are significant ($p < .05$), and the horizontal dashed line indicates the number of years that must be included ($N = 19$) for the trend to be significant from all vantage points. The open circles identify combinations of onset and duration that have been used to identify the "hiatus" by articles in the corpus. Multiple articles may contribute to a given circle. **B**: Level of statistical significance for trends (GISS, K/decade) as a function of vantage year and the number of years included in the computation of the trend as in panel A. Trends that are clearly non-significant ($p > .10$) are shown in beige, those that approach significance ($.05 < p < .10$) are shown in shades of gray, and significant trends ($p < .05$) are shown in shades of terracotta.

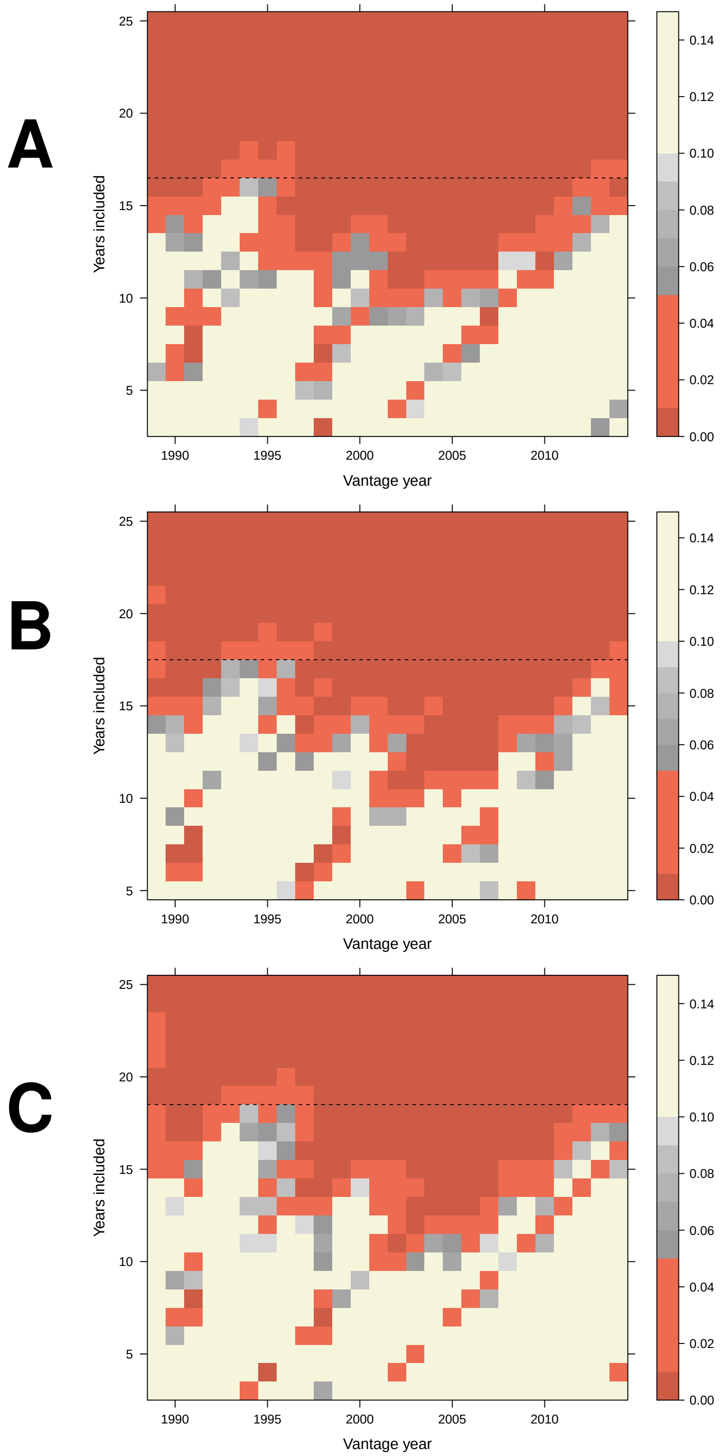

Figure S4. : All panels show the level of statistical significance for trends (K/decade, based on data of Cowtan & Way, 2014) as a function of vantage year and the number of years included in the computation of the trend. Trends that are clearly non-significant ($p > .10$) are shown in beige, those that approach significance ($.05 < p < .10$) are shown in shades of gray, and significant trends ($p < .05$) are shown in shades of terracotta. Panels differ with respect to modeling of autocorrelations in the time series. **A**: Ordinary least squares analysis. **B**: Generalized least squares analysis with autocorrelation among residuals modeled by an AR1 process as recommended by the IPCC (Hartmann et al., 2013). **C**: Ordinary least squares analysis on a prewhitened time series. See text for details on prewhitening.
